# Present Status, Limitations and Future Directions of Treatment Strategies Using Fucoidan-Based Therapies in Bladder Cancer

**DOI:** 10.3390/cancers12123776

**Published:** 2020-12-15

**Authors:** Yasuyoshi Miyata, Tomohiro Matsuo, Kojiro Ohba, Kensuke Mitsunari, Yuta Mukae, Asato Otsubo, Junki Harada, Tsuyoshi Matsuda, Tsubasa Kondo, Hideki Sakai

**Affiliations:** Department of Urology, Graduate School of Biomedical Sciences, Nagasaki University, Nagasaki 852-8501, Japan; tomozo1228@hotmail.com (T.M.); ohba-k@nagasaki-u.ac.jp (K.O.); ken.mitsunari@gmail.com (K.M.); ytmk_n2@yahoo.co.jp (Y.M.); a.06131dpsc@gmail.com (A.O.); harada-junki@nagasaki-u.ac.jp (J.H.); matsudatsuyoshi9251@gmail.com (T.M.); t-udonko@nagasaki-u.ac.jp (T.K.); hsakai@nagasaki-u.ac.jp (H.S.)

**Keywords:** fucoidan, molecular mechanisms, combination therapy, nanoparticles, bladder cancer

## Abstract

**Simple Summary:**

Prognosis of bladder cancer patients is often poor despite various intensive treatments are performed. Therefore, many investigators pay attention to the efficacy of natural product-based treatments to avoid additional adverse events in these patients. Here, we review the anti-cancer effects of fucoidan-based treatments and the protective effects against cancer-related disorders and cisplatin-induced toxicities.

**Abstract:**

Bladder cancer (BC) is a common urological cancer, with poor prognosis for advanced/metastatic stages. Various intensive treatments, including radical cystectomy, chemotherapy, immune therapy, and radiotherapy are commonly used for these patients. However, these treatments often cause complications and adverse events. Therefore, researchers are exploring the efficacy of natural product-based treatment strategies in BC patients. Fucoidan, derived from marine brown algae, is recognized as a multi-functional and safe substrate, and has been reported to have anti-cancer effects in various types of malignancies. Additionally, in vivo and in vitro studies have reported the protective effects of fucoidan against cancer-related cachexia and chemotherapeutic agent-induced adverse events. In this review, we have introduced the anti-cancer effects of fucoidan extracts in BC and highlighted its molecular mechanisms. We have also shown the anti-cancer effects of fucoidan therapy with conventional chemotherapeutic agents and new treatment strategies using fucoidan-based nanoparticles in various malignancies. Moreover, apart from the improvement of anti-cancer effects by fucoidan, its protective effects against cancer-related disorders and cisplatin-induced toxicities have been introduced. However, the available information is insufficient to conclude the clinical usefulness of fucoidan-based treatments in BC patients. Therefore, we have indicated the aspects that need to be considered regarding fucoidan-based treatments and future directions for the treatment of BC.

## 1. Introduction

Bladder cancer (BC) is a common malignancy of the urinary system. Generally, the prognosis of BC is relatively good if cancer cells do not invade the muscle layer and disseminate to lymph nodes or distant organs. On the other hand, outcomes for patients with advanced forms of this disease, including muscle invasion and/or metastasis, is poor despite various treatments such as radical cystectomy, chemotherapy, immune therapy, and radiotherapy. Moreover, most therapies for such advanced BC lead to a decrease in the quality of life (QoL) owing to complications and adverse events. Therefore, information on additional therapeutic strategies that use safe and low-cost agents is important for maintaining the QoL and improving prognosis in patients with advanced BC. Consequently, many investigators have paid special attention to the preventive effects against adverse events and anti-cancer properties of natural products in the treatment of BC [1,2,3,4].

Fucoidan is a marine sulfated carbohydrate derived from marine brown algae. It is a heparin-like molecule with an α-1,3-linked fucose and an α-1,4-linked fucose with branches attached at the C2 position [5]. It is known to have various biological activities, including antibacterial, anti-inflammatory, antioxidant, and immunomodulatory effects [6,7,8]. Additionally, fucoidan has been favored owing to its low toxicity in vivo, including in humans [9,10]. Furthermore, there is a general agreement that fucoidan exerts anti-cancer effects by regulating tumor growth, cancer cell apoptosis, invasion, metastasis, cell cycle, tumor angiogenesis, and immune reactivities in various types of malignancies [5,11,12,13,14,15]. Numerous factors and molecules have been reported in in vivo and in vitro studies as part of the molecular mechanisms underlying the anti-cancer properties of fucoidan. These include the phosphatidylinositol-3-kinase (PI3K)/AKT signaling pathway in hepatocellular carcinoma and colon cancer [16,17], Bcl-2 family in lung cancer [18], caspases in breast cancer [19], cyclins in cervical cancer [20], and cytochrome c in osteosarcoma [21]. Excellent reviews are available on the structure, biological activity, and interactions of fucoidan under various physiological and pathological conditions [6,8,10,14]. On the other hand, there are only a few systematic reviews depicting the pathological significance of fucoidan and the molecular mechanisms of its anticancer effects in BC, although some previous studies have shown and discussed these factors in original articles. 

Various pathological steps are necessary for tumor growth, invasion, and metastasis in solid tumors. Furthermore, many factors are associated with these processes, such as cancer cell proliferation, apoptosis, cell cycle, cell migration, invasion, and angiogenesis. In addition, at the molecular level, numerous cancer-related molecules modulate these pathological processes as stimulators or suppressors. Importantly, several natural foods and their extracts can alter such malignant processes at the molecular level in BC, as observed in in vivo and in vitro studies [22,23,24,25]. Thus, understanding the influence of fucoidan on malignant behavior and its regulatory mechanisms at the molecular level is essential to formulate treatment strategies in patients with BC. Various treatment strategies using nanoparticles with fucoidan have been reported in various types of malignancies. In this review, we highlight and discuss the following aspects: (1) pathological roles of fucoidan in malignancies, (2) effects of combination therapies of fucoidan and conventional anti-cancer agents, (3) trials of nanoparticles including fucoidan, (4) differences in biological and pharmacological activities of fucoidans according to species and molecular weight, and (5) protective effects of fucoidan in cancer-related disorders. Lastly, we discuss the future directions and limitations of fucoidan-based therapies in malignancies, including BC.

## 2. Biological Effects of Fucoidans in Bladder Cancer Cells

### 2.1. Effect on Cell Proliferation and Tumor Growth

One of the most important determinants of tumor growth and development is the regulation of cell survival, which comprises cell proliferation and cell death. In fact, several studies have focused on the relationship between fucoidan and tumor growth, cancer cell proliferation, and/or apoptosis in BC [26,27,28,29,30]. To the best of our knowledge, the anti-tumor effects of fucoidan in BC, including suppression of cancer cell proliferation and induction of apoptosis, were first reported in two papers published in 2014 [26,27]. In both the studies, the fucoidan used was obtained from the same company (Sigma-Aldrich Chemical Co., St. Louis, MO, USA) and the studies were performed by the same research group in Korea. However, in one of the studies, the researchers used 5637 cells (originating from grade 2 carcinoma) and T24 cells (undifferentiated grade 3 carcinoma), whereas in the other, they used T24 cells [26,27]. Cho et al. [26] reported that 5637 cell viability was inhibited in a concentration-dependent manner when cultured in a standard medium containing various concentrations (0–400 μg/mL) of fucoidan for 24 h. Similarly, the other report showed that fucoidan inhibited T24 cell viability in a concentration- and time-dependent manner [27]. However, it should be noted that the anti-proliferative effects of fucoidan, when examined in detail, were different between them, despite the fact that the source of fucoidan was the same and cell viability was measured using the same method (3-(4,5-dimethyl-2-thiazolyl)-2,5-diphenyl-2H tetrazolium; MTT assay). Briefly, although 5637 cell viability was inhibited by ≥50 μg/mL of fucoidan for 24 h, T24 cell viability was inhibited by ≥100 μg/mL of fucoidan for 48 h, but not by 50 μg/mL [26,27]. Furthermore, in 2017, the same study group showed that treatment of 5637 cells with 25 μg/mL of fucoidan for 24 h (purified from *Fucusvesiculosus* (purchased from Sigma-Aldrich Chemical Co., St. Louis, MO, USA) significantly inhibited cell viability (measured via MMT assay) [30]. Thus, the anti-proliferative effect depended on the type of cancer cells, and it was speculated that high-grade BC was able to tolerate fucoidan. Subsequently, such fucoidan-induced anti-proliferative effects in 5637 and T24 cells have been reported in other studies [28,30]. Additionally, a dose-dependent anti-proliferative effect (50–150 μg/mL) of fucoidan was observed in other human bladder cancer cells (EJ cells) [29].

On the other hand, there are no in vivo studies regarding the anti-proliferative effects of fucoidan on BC. However, Chen et al. [28] reported that low-molecular-weight fucoidan (LMWF) inhibited tumor growth in vivo. Briefly, when 80, 160, and 300 mg/kg/day LMWF (molecular weight was mainly 760 Da) was orally administered for 30 days to BALB/c nude mice that were injected with T24 cells, the tumor size and weight decreased significantly in mice treated with 160 and 300 mg/kg/day LMWF but not in those administered a fucoidan dose of 80 mg/kg/day [28].

### 2.2. Effect on Apoptosis

Like the anti-proliferative effects, the pro-apoptotic effects of fucoidan were first reported in an in vitro study in 2014 [27]. In this study, the authors evaluated apoptosis in T24 cells using three methods, namely, nuclear morphological change, DNA fragmentation, and annexin V staining. Increase in nuclear chromatin condensation, DNA fragmentation, and annexin V-stained cells was observed in a dose-dependent manner after treatment with various concentrations of fucoidan (50, 100, and 150 μg/mL) for 48 h [27]. The number of apoptotic cells measured by the percentage of annexin V-positive/propidium iodide-negative cells in cells treated with 100 and 150 μg/mL fucoidan (approximately 20 and 26%, respectively) were remarkably higher than that in control cells (0 μg/mL, approximately 2%) [27]. Additionally, dose-dependent pro-apoptotic activity was observed using similar methods in other types of EJ cells [29]. On similar lines, another study in 2017 investigated the relationship between fucoidan and apoptosis in in vitro studies using 4,6-diamidino-2-phenylindole (DAPI) staining and flow cytometry in 5637 cells [30]. In this study, BC cells were treated with 0, 10, 25, or 100 μg/mL of fucoidan for 24 h; nuclear fragmentation and chromatin condensation were found to increase in a concentration-dependent manner. Furthermore, flow cytometry results showed that the percentage of cells with sub-G1 DNA content, which is a parameter of apoptosis, was also increased in a concentration-dependent manner (0 μg/mL = 2.97%, 10 μg/mL = 3.17%, 25 μg/mL = 6.47%, 50 μg/mL = 19.87%, and 100 μg/mL = 40.12%) [30]. Thus, the pro-apoptotic effect of fucoidan was confirmed in BC cells using various methods. However, in vivo studies regarding these effects of fucoidan, including LMWF, have not been performed yet. Moreover, there are no data on the relationship between fucoidan and non-apoptotic cell death, such as necrosis and ferroptosis in BC, despite their correlation being studied in other malignancies [21].

### 2.3. Effect on Cell Migration and Invasion

To our knowledge, only one study has investigated the relationship between fucoidan and BC cell migration/invasion [26]. This study used scratch assay and cell invasion assay to show that fucoidan inhibited the migration and invasion of two different BC cell lines (5637 and T24 cells) [26]. Interestingly, the healing area (% of control) in 5637 cells treated with 100 μg/mL of fucoidan after 24 h was 45 ± 5.05%, similar to that in T24 cells treated under similar conditions (46 ± 5.26%) [26]. Additionally, the cell invasion assay showed that the proportion of invasive cells (% of control) in 5637 cells was similar to that in T24 cells (15 ± 7.53 and 17 ± 6.12%, respectively) under similar culture conditions (100 μg/mL fucoidan after 24 h) [26]. This study demonstrated that the inhibitory effects of fucoidan on cell migration and invasion might be dependent on the malignant potential of BC cells [26]. In addition, there was a report that LMWF inhibited cell migration and invasion of T24 cells in a dose-dependent manner [28]. However, it should be noted that these analyses were performed under hypoxic conditions of 1% O_2_.

### 2.4. Effects on Angiogenesis

Metastasis is the most important predictor of survival, and angiogenesis is recognized as an important step in disseminating cancer cells from the primary tumor mass. Therefore, many investigators have reported the relationship between tumor angiogenesis and crude fucoidan or fucoidan extracts in various types of cancers [13,31,32]. For example, abalone glycosidase-digested fucoidan extracts derived from the seaweed, *Mozuku* (*Cladosiphon novae-caledoniae Kylin*), inhibited vascular tube formation, including the number, total length, and total area of tubes in human cervical cancer cells (HeLa cells) in an in vitro study [31]. 

Unfortunately, the relationship between fucoidan and angiogenesis in BC is not fully understood. One study investigated the anti-angiogenic activities of LMWF using in vivo and in vitro studies [28], wherein LMWF suppressed capillary tube-formation in human umbilical vein endothelial cells (HUVECs) under hypoxic conditions. Notably, such anti-angiogenic activity was not observed under normoxic conditions, implying that LMWF may suppress only hypoxia-induced angiogenesis. Generally, hypoxia in the tumor microenvironment is one of the most representative characteristics of solid tumors and plays crucial roles in malignant potential, tumor development, and outcomes in various types of malignancies [33,34,35]. Consequently, it is possible that the specific anti-angiogenic activity of LMWF under hypoxic conditions is advantageous for increasing anti-cancer effects and decreasing adverse events in cancer patients because it leads to stronger biological effects of fucoidan in cancer tissues compared to that in normal tissues. On the other hand, more detailed information, especially the correlation of LMWF with cell proliferation and migration of endothelial cells, is essential to discuss its anti-cancer effects via regulation of angiogenesis in malignant tumors. Chen et al. [28] also analyzed the microvessel density after staining using the anti-CD31 antibody in matrigel plug and injecting T24 cells in nude mice (BALB/c). Results showed that the amount of CD31 in matrigel and tumor tissues under stimulation by vascular endothelial growth factor (VEGF) was suppressed in a dose-dependent manner by the administration of LMWF (matrigel plug: 0, 25, 50, and 75 μg, T24 tumors: 0, 80, 160, and 300 μg/kg/day). Specifically, CD31-positive vessel density under VEGF stimulation in matrigel plugs with 50 and 75 μg of fucoidan was significantly lower than that in the control group plugs (0 μg). On the other hand, CD31-stained capillaries were remarkably decreased in tumor tissues in mice treated with 160 and 300 μg/kg/day of fucoidan.

Thus, the cell lines used and the species, types, and doses of fucoidans are closely associated with the biological effects of fucoidans in BC. A summary of the pathological roles of fucoidans in BC cells according to these parameters is given in Table 1. 

## 3. Molecular Mechanisms of Fucoidans Underlying Their Anti-Cancer Effects in Malignancies

### 3.1. Anti-Cancer Cell Growth and Survival

Cho et al. [26] reported that fucoidan inhibited the proliferation of 5637 cells (Cho 2014). They speculated that PI3K/AKT signaling played a crucial role in this fucoidan-induced anti-proliferative effect because fucoidan-induced activation of AKT was inhibited by a PI3K-specific inhibitor, and subsequent blockage of AKT signaling led to the inhibition of fucoidan-induced inhibitory effect [26]. 

A previous study reported fucoidan-induced apoptosis of T24 cells in a concentration-dependent manner [27]. In this study, the detailed molecular mechanisms of fucoidan-induced apoptosis in BC cells were investigated. The authors discovered that: (1) the initiator of the extrinsic apoptotic pathway (caspase-8) and the intrinsic apoptotic pathway (caspase-9) were associated with fucoidan-induced apoptosis; (2) the Fas/Fas ligand (FasL) system, which belongs to the extrinsic apoptotic pathway, is a key signaling transduction pathway in this system; (3) activation of caspase-3 and cleavage of the pro-form poly(ADP-ribose) polymerase (PARP) protein to its inactive form led to its pro-apoptotic activity; (4) fucoidan-induced apoptosis was positively associated with increase in Bax and decrease in Bcl-2 (increase in the Bax/Bcl-2 ratio, characterizing the intrinsic apoptotic pathway); (5) fucoidan treatment resulted in downregulation of inhibitors of apoptosis (IAP) family numbers, such as XIAP, cIAP-1, and cIAP-2, and full-length Bid (a BH3-only protein from the Bcl-2 family); and (6) fucoidan-induced apoptosis via regulation of mitochondrial function, such as, a significant decrease in cytochrome c in the mitochondria and loss of the mitochondrial membrane potential [27]. Thus, treatment with fucoidan induced apoptosis via complex mechanisms in T24 cells. A summary of fucoidan-induced changes of tumor growth- and/or apoptosis-related molecules was showed in Table 2.

Several investigators have shown that control of the cell cycle by fucoidan also plays an important role. Briefly, fucoidan induced G1 phase cell cycle arrest in 5637 cells via the upregulation of p21Waf1 expression and suppression of cyclins and CDK expression [26]. Additionally, these phenomena were negated when AKT signaling was blocked [26]. Therefore, the authors concluded that fucoidan had a significant inhibitory effect on tumor growth, followed by G1-phase-associated upregulation of p21Waf1 expression and suppression of cyclins and CDK expression in BC [26]. Similarly another study showed that the proportion of cells in the G1 phase in T24 cells treated with control medium (0 μg/mL of fucoidan) was 34.2%, and those in cells treated with 50, 100, and 150 μg/mL of fucoidan were 52.1%, 61.7%, and 67.8%, respectively [27]. Thus, the author corroborated the notion that fucoidan modulated the cell cycle in BC cells. Furthermore, the molecular mechanisms of fucoidan-induced cell cycle arrest in the G1 phase and decreased expression of cyclin D1, cyclin E, and CDK were reported in this study [27]. These results were supported by the findings of the above-mentioned report [26]. Additionally, Park et al. [27] demonstrated that the expression of CDK inhibitor p21 was increased at the transcriptional and translational levels in T24 cells treated with fucoidan. 

Furthermore, Park et al. [27]. investigated the relationship between the phosphorylation of retinoblastoma (Rb) and the transcription factors E2Fs in T24 cells because Rb is an important checkpoint in the G1 phase [36]. They found that pRb expression was decreased after fucoidan treatment in a time-dependent manner, and a strong increase in the association of pRB and E2F-1 as well as E2F-4 post fucoidan treatment in T24 cells was observed [27]. The authors concluded that fucoidan inhibits the release of E2Fs proteins from pRb in T24 cells. Likewise, the same study group performed similar research in another kind of human bladder cancer (RJ) cells, and reported that fucoidan induced G1 arrest through downregulation of pRb via increased binding of pRb to E2Fs (1 and 4) [27]. 

Summary of fucoidan-induced changes of cell-cycle-related molecules was showed in Table 3. 

### 3.2. Anti-Invasive and Migration Effects

Cancer cell migration and invasion are important steps in cancer cell dissemination into surrounding tissues, lymph nodes, and distant organs. In fact, muscle invasion is closely associated with dismal prognosis in patients with BC [37,38]. Although the invasive step of BC is regulated by many molecules, matrix metalloproteinases (MMPs) are considered to be one of the most important stimulators in BC tissues [39,40,41]. Among MMP members, the pathological significance and prognostic roles of MMP-2 and -9 have been investigated most widely in many types of cancers [42,43,44]. In 2005, Ye et al. [31]. reported that enzyme-digested fucoidan extracts inhibit cell invasion via the downregulation of MMP-2 and -9 in human fibrosarcoma (HT1080) cells. Subsequently, other investigators reported that a mixture of fucoidan and vitamin C suppressed HT1080 cell invasion via suppression of MMP-2 and -9 activities [45]. Furthermore, other investigators showed that the sulfated fucoidan (98% purity) obtained from *Sargassum fusiforme* suppressed cell migration and invasion of hepatocellular cancer cells (HCC SMMC-7721, Huh7, and HCCLM3 cells), and the decreased expression of MMP-2 was speculated to be associated with this outcome [11]. Similar anti-cancer effects of fucoidan via the regulation of MMP-2 have also been reported in lung cancer cells (Lee 2012) [46]. Thus, fucoidan is speculated to play crucial roles in cell migration and invasion via regulation of MMP-2 and -9 in several malignancies. Similar findings were reported in BC cells (5637 cells) [26]. This study also showed that fucoidan-related MMP-9 expression was mediated by activator protein (AP)-1 and NF-κB binding activity, and that treatment with wortmannin, a PI3K-specific inhibitor, abolished tumor suppressive effects by regulating MMP-9, NF-κB, and AP-1 in fucoidan-treated cells [26]. Lastly, the authors concluded that activation of AKT was closely associated with BC cell migration and invasion via inhibition of MMP-9 expression through reduction of AP-1 and NF-κB activities [26]. However, to our knowledge, there is no other report on the molecular regulatory mechanisms of fucoidan on MMP-2 and -9 expression in BC. Correspondingly, fucoidan suppressed cancer cell migration and invasion in human lung cancer cells (A549 cells) via inhibition of MMP-2, wherein blocking of the ERK1/2 and PI3K-AKT-mTOR pathways was associated with the MMP-2-related anti-cancer effects of fucoidan [46]. 

Thus, several reports have described the regulatory mechanism of fucoidan on MMP-2 and -9 at the molecular level in malignant cells. However, these results are not adequate to discuss fucoidan-based therapeutic strategies in patients with cancer. Fucoidan-induced changes in the expression levels and activities of other MMP members in malignant cells are not fully understood, even though MMPs other than MMP-2 and -9 also play important roles in malignant aggressiveness and prognosis in various types of cancers, including BC [47,48,49,50,51]. 

### 3.3. Role of Oxidative Stress

Oxidative stress plays an important role in the regulation of various biological activities, including cell survival and metabolism under physiological and pathological conditions [52,53]. Oxidative stress induces excessive production of reactive oxygen species (ROS), and increased intracellular ROS damages cell components such as proteins, lipids, and DNA [54]. Additionally, elevated ROS production is closely associated with malignant aggressiveness via regulation of various cancer-related molecules in many types of cancers, including BC [55,56,57]. However, fucoidan was reported to induce apoptosis via upregulation of intracellular ROS production in BC cells (5637 cells) [30]. This study also showed that the PI3K/AKT pathway and telomerase activity were associated with such fucoidan-induced apoptotic function.

## 4. Combination of Fucoidan and Conventional Chemotherapeutic Agents

Treatments with cisplatin (CDDP) and gemcitabine (GEM) are recognized as standard chemotherapeutic regimens in patients with advanced/metastatic BC [58]. Additionally, taxanes, including paclitaxel (PTX) and docetaxel (DTX), are often used as second- or third-line of therapy for platinum-resistant BC [59,60]. Some investigators have paid special attention to combination therapies of fucoidan and conventional anti-cancer agents, including CDDP, GEM, and taxanes. However, unfortunately, there is no report on anti-cancer effects of such a combination therapy on BC yet. Therefore, we have described the anti-cancer effects of combination therapy of fucoidan and these chemotherapeutic agents in other types of malignancies. 

### 4.1. Cisplatin

Several investigators have shown that crude fucoidan enhances the cytotoxic effects of CDDP in several cancer cell types. For example, in head and neck squamous cell carcinoma cells, a combination of crude fucoidan (derived from *Fucusvesiculosus*) and CDDP showed synergistic anti-proliferative effects in all tested cancer cell lines (H103, FaDu, and KB cells) and synergistic pro-apoptotic effects in H103 and KB cells [61]. Interestingly, although regulation of ROS production and cell cycle were associated with a part of these fucoidan-induced anti-cancer effects, the influence of these cancer-related mechanisms was different among the three cancer cell lines. Briefly, ROS production was increased in H103 cells, was not significantly changed in FaDu cells, and was decreased in KB cells [61]. The cell cycle was arrested in the S/G2 phase in H103 and FaDu cells and in the G1 phase in KB cells. Finally, the authors speculated that KB cells showed the most sensitivity to the combination of fucoidan and CDDP treatment [61]. Similarly, the combination of fucoidan with CDDP, doxorubicin, and PTX displayed enhanced cytotoxic effects in breast cancer cells (MCF-7 cells) via regulation of apoptosis and cell cycle [62]. This study also showed that fucoidan did not have harmful effects, including apoptosis in normal cells (MCF-12 cells) [62]. Based on these findings, the authors of these two studies concluded that fucoidan was a promising candidate for combination therapy with conventional therapeutic agents in patients with breast cancer [61,62]. Moreover, in lung cancer cells (LLC1 cells), sequential treatment with CDDP and fucoidan was reported to have stronger anti-cell-growth effect than that with CDDP alone, through upregulated caspase-3 and PARP activities [63]. In addition to the above-mentioned in vitro studies, this study showed that fucoidan increased CDDP-induced cytotoxicity in an in vivo lung cancer model using LLC1-bearing C57BL/6 mice [63]. Briefly, the tumor volume in C57BL/6 mice subcutaneously injected with LLC1 cells, after the sequential treatment with CDDP (intraperitoneal injection of 1.0 mg/kg at day 1) followed by fucoidan (oral intake of 15 mg/kg/day for the duration of the treatment period), was significantly lower than that in mice treated with CDDP alone [63]. Thus, crude fucoidan has additional and synergistic anti-cancer effects with CDDP in various types of cancers.

Similarly, there was a report that extract from the seaweed *Cladosiphon novae-caledoniae* consisting of a digested small molecular weight fraction (72%; <500 Da) and a non-digested fraction (less than 28%; peak = 800 kDa) enhanced the anti-cancer effects of CDDP via inhibitory effects on cell growth (using 200 and 400 μg/mL fucoidan extract for 48 h) and the pro-apoptotic activity (using 200 μg/mL fucoidan extract for 24 and 48 h) in breast cancer cells (MDA-MB-231 and MCF-7 cells) [64]. Furthermore, in recent years, oligo-fucoidan (molecular weight: 92.1%; 500–800 Da) has been reported to promote the cytotoxic activity of CDDP in colon cancer cells [12]. Briefly, in primary C6P2-L1 cell lines derived from colorectal cancer patients, the number of apoptotic cells in the group treated with a combination of CDDP and oligo-fucoidan was significantly higher than those in groups treated with CDDP alone or oligo-fucoidan alone, and upregulation of PARP cleavage and caspase-3 activation were associated with these results [12]. This study also showed that oligo-fucoidan enhanced the anti-tumor effects of CDDP in colorectal cancer cells in vivo. Briefly, in a xenograft model with subcutaneous injection of HCT 116 cells, tumor volume in the group with a combination treatment of CDDP and oligo-fucoidan was significantly lower than that in the group with CDDP treatment alone [12]. Notably, such additional anti-tumor growth effects of fucoidan with CDDP were detected in p53^+/+^ tumors only and not in p53^−/−^ tumors [12]. 

### 4.2. Gemcitabine

To our knowledge, there are very few studies on the cooperative effects of fucoidan and GEM in cancer cells. In one such study, the anti-growth effects of a combination of GEM and fucoidan extracts derived from *Undaria pinnatifida* and *Fucusvesiculosus* were analyzed in various types of malignant cell lines [65]. Antagonistic interactive effects of GEM were observed with fucoidan derived from *Undaria pinnatifida* in breast cancer cells (HCC-38 cells) and tongue squamous cell carcinoma cells (CAL-27 cells), and with that from Fucusvesiculosus in HCC-38 cells, CAL-27 cells, ovarian cancer cells (SKOV-3), and melanoma cells (HS294T cells) [65]. Thus, several fucoidans may affect the biological and pharmacological activities of GEM in some malignant cells; however, their efficacy is not clear because there has been no in vivo study. 

On similar lines, there was a report that combination therapy with fucoidan and GEM had additive and synergistic anti-tumor effects in uterine sarcomas and carcinosarcoma cells [61]. In this study, fucoidan from *Undaria pinnatifida* had an additive anti-tumor effect in combined treatment with GEM and fucoidan in ESS-1 cells (endometrial stromal sarcoma cells) and SK-UT-1 cells (carcinosarcoma cells), and the synergistic effect was detected in SK-UT-1B cells (carcinosarcoma cells) [61]. Additionally, this study showed that the combination of GEM and fucoidan displayed significant additional pro-apoptotic effects in ESS-1 cells; however, such a significant additive effect was not observed in carcinosarcoma cells (SK-UT-1 and SK-UT-1B cells) [61]. Contrastingly, there was hardly any merit of this combination therapy in uterine leiomyosarcoma cell line (MES-SA cells) [63]. Such information is important in understanding the specificity of combination therapy of GEM and fucoidan according to types of malignant cells and their limitations on anti-tumor effects.

### 4.3. Taxanes

Mathew et al. [65]. reported the anti-proliferative effects of a combination of crude fucoidans and various conventional chemotherapeutic agents, including PTX. In this study, two different fucoidan extracts derived from *Undaria pinnatifida* and *Fucusvesiculosus*, were investigated, and PTX demonstrated synergistic growth-inhibitory effects in combination with both the fucoidans in many types of cancer cell lines (cervical cancer: HeLa and SiHa, ovarian cancer: TOV-112D and SKOV-3, endometrial carcinoma: HEC-1A and Ishikawa, melanoma: HS294T, tongue squamous cell carcinoma: CAL-27, and prostate cancer: PC-3) [61]. Furthermore, the same study group confirmed the anti-tumor effects of these combined treatments in in vivo studies using human cancer orthotopic mouse models [66]. In contrast to the results of in vitro studies, combination of PTX and fucoidan extract derived from *Undaria pinnatifida* or *Fucusvesiculosus* showed no significant effects on tumor growth in human ovarian cancer orthotopic models with SKOV-3 as well as TOV-112D cell lines [66]. However, surprisingly, these combinations of fucoidans and PTX significantly enhanced tumor growth in mouse models of breast cancer, using MCF-7 and ZR-75 cells [66]. Although there is no similar study in BC, such information is extremely important to plan further studies on the anti-cancer effects of a combination treatment of fucoidans and PTX in BC. On the other hand, fucoidan extracts composed of LMWF (<50 Da) and HWMF (800 kDa) enhanced the anti-proliferative and pro-apoptotic effects of PTX in two breast cancer cell lines (MCF-7 and MDA-MB-231 cells) [67]. This study also reported that increase in oxidative stress was a crucial process in the anti-cancer effects of fucoidan extract and chemotherapeutic agents, including PTX, because enhanced intracellular ROS production and reduced antioxidant levels were detected in this process [67]. These findings support the hypothesis that regulation of oxidative stress may modulate the anti-cancer effects of fucoidan-based chemotherapy in cancer patients. In Table 4, we showed the summary of increased anti-cancer effects of fucoidan combined with CDDP, GEM, or PTX.

## 5. Nanoparticles with Fucoidans

There is a general agreement that anti-cancer therapy employing a drug delivery system using nanoscale drug carriers is a useful and promising method in various types of cancers [68]. Accordingly, many investigators have focused on treatment strategies using fucoidan-based nanoparticles in various malignancies [69,70,71]. However, information on the anti-cancer effects of fucoidan-based nanoparticles in BC cells has not been reported yet. On the other hand, anti-cancer effects of fucoidan-based nanoparticles with CDDP, GEM, and taxans, which have been recognized as standard anti-cancer agents for BC, have been reported in various types of malignant cells. Therefore, in this section, we present this information to discuss the possibility of a promising treatment strategy for BC. 

Hwang et al. [68] reported that CDDP–fucoidan (derived from *Fucusvesiculosus*) nanoparticles had stronger anti-cancer effects than CDDP alone, wherein the nanoparticles increased the anti-cancer immunity and cytotoxic effects in human ileocecal adenocarcinoma cells (HCT-8 cells). Interestingly, although various CDDP–fucoidan nanoparticles were prepared using different concentrations of CDDP (0.5, 1.5, 2.0, and 4.0 mg/mL) and fucoidan (2.5, 5.0, 7.5, and 10.0 mg/mL), nanoparticles made using 2 mg CDDP and 10 mg fucoidan exhibited the strongest anti-cancer effects [68]. 

Likewise, the cytotoxic effects of GEM drug delivery using nanoparticles made from fucoidan and chitosan have been reported [72]. Briefly, cytotoxic effects on breast cancer increased by 25% upon using GEM-loaded nanoparticles (around 115–140 nm in size) based on fucoidan- and chitosan-origin polymers [72]. 

The cytotoxic effects of DTX-encapsulated fucoidan-polymeric micelles poly(lactic-co-glycolic acid) nanocarriers against triple-negative breast cancer cells were reported earlier in 2020 [73]. In that study, fucoidan was derived from *Fucusvesicululos*, and MDA-MB-231 cells were used. The authors concluded that the nanoparticles effectively exerted better anti-cancer effects and were recognized as a competent drug delivery system [73]. Moreover, other investigators showed the synergistic effects of DTX and fucoidan in multifunctional nanoparticles encapsulated in green tea polyphenol and low-dose DTX within fucoidan-based nanoparticles against prostate cancer [74]. We concur with the concept of their treatment regimen using low-dose cytotoxic agents because we also reported the safety of chemotherapeutic regimens using low-dose PTX in patients with BC [75,76]. Correspondingly, a study group focused on new cancer treatment strategies using fucoidan nanoparticles loaded with PTX [77,78]. Briefly, the authors analyzed the loading efficiency and release patterns of fucoidan nanoparticles with curcumin and PTX to investigate their potential as promising anti-cancer agents [77,78]. We have a keen interest regarding the anti-cancer effects and safety of patients with BC because, in addition to fucoidan, these studies used natural products, including green tea polyphenol and curcumin, which have been widely reported to suppress tumor growth, progression, and treatment resistance in BC [1,79,80,81]. 

## 6. Protection against Cancer-Related Disorders and Adverse Events

In the earlier section, we focused on the biological and pharmacological roles of fucoidan and its anti-cancer effects in BC. However, in the care and treatment of patients with BC, especially in advanced/metastatic disease, management of tumor-related cachexia and/or treatment-induced decline in physical strength is also necessary to improve the prognosis and maintain the QoL of patients. In fact, in cases of unresectable advanced or recurrent colorectal cancer, patients treated with 4.05 g fucoidan for six months from the initial day of chemotherapy (FOLFOX or FOLFIRI) reported significantly reduced frequency of fatigue compared to those treated with chemotherapy alone [82]. In this section, we present the protective effects of fucoidan against cancer-related disorders and adverse events, especially with respect to skeletal muscle loss and CDDP-induced adverse events.

Skeletal muscle atrophy is one of the most representative features of cancer cachexia, and is often observed in cancer patients undergoing chemotherapy [83,84]. Skeletal muscle atrophy leads to a decrease in QoL owing to a reduction in social activity and exercise, along with clinical problems including poor tolerance to cancer therapy [85]. Therefore, many investigators have focused on the prevention of chemotherapy-induced anorexia, including skeletal muscle atrophy, via various nutritional supplements or medications [86,87,88]. In the case of BC, several investigators have opined that sarcopenia, which is defined as the degenerative and systemic loss of skeletal muscle mass, plays an important role in the prognosis and survival of patients treated with radical cystectomy, systematic chemotherapy, and radiotherapy [89,90,91]. Consequently, several chemical agents and natural products have been investigated to assess whether they suppress the chemotherapy-induced skeletal muscle atrophy in BC-bearing mice, including, anamorelin, a ghrelin receptor agonist, and magnolol, isolated from the Chinese herb, *Magnolia officinalis* [92,93]. Similarly, fucoidan was reported to inhibit tumor- and chemotherapy-induced skeletal muscle atrophy in BC-bearing mice [86]. Briefly, muscle atrophy in orthotopic mice transplanted with T24 cells and treated with a combination regimen of CDDP and GEM was remarkably suppressed by LMWF (mainly molecular weight was 760 Da) derived from *Sargassum hemiphyllum* [86]. The authors also showed that favorable control of inflammation, muscle proteolysis, and protein synthesis by myostatin/activin A/FoxO3/MAFbx/MuRF-1 cascade, NF-κB, and IGF-1 played crucial roles in the mitigation of chemotherapy-induced toxicity [86]. Additionally, this study demonstrated that fucoidan suppressed intestinal damage and function in a similar animal model [86]. Lastly, they speculated that LMWF is a promising and useful nutritional supplement and chemotherapeutic adjuvant for minimizing chemotherapy-induced toxicities in patients with BC, and we agree with their opinion.

As mentioned above, CDDP is a key drug in the treatment of BC. However, CDDP causes relatively severe adverse events in the gastrointestinal tract and kidneys. LMWF extracted from *Undaria pinnatifida* inhibits the chromic CDDP-induced weight loss and delayed gastrointestinal motility in a rat model [94]. Furthermore, in an in vitro study using proximal tubule epithelial (TH-1) cells, fucoidan suppressed CDDP-induced apoptosis and cell-cycle arrest via its anti-oxidative effects, including decreased ROS accumulation and excessive ER stress [95]. Accordingly, the authors suggested that fucoidan may be useful in protecting renal function in patients with cancer, who were treated with CDDP [95]. 

## 7. Issues Worth Considering and Future Direction of Fucoidan-Based Therapies

### 7.1. Points to Be Aware of Regarding Discussion of Fucoidan-Based Treatments

While discussing the anti-cancer effects and clinical usefulness of fucoidan in cancer therapy, special attention must be paid to its species, extraction methods, administration methods, and harvesting seasons. Briefly, bioactivities, including the anti-cancer effects of fucoidan, depend on these internal and external factors [10,15,63,96,97,98].

For example, although 50% cell proliferation of lung cancer cells (A549 cells) was inhibited by treatment for 48 h with 700 μg/mL fucoidan extracted from *Undaria pinnatifida* [97], a similar anti-cancer effect in A549 cells was shown by treatment for 48 h using only 100 μg/mL fucoidan extracted from *Fucusvesiculosus* [63]. Additionally, there is a report suggesting that fucoidans from *Macrocystis pyrifera* and *Undaria pinnatifida* at concentrations of 5–100 μg/mL display inhibitory effects on neutrophil apoptosis; however, this effect, in fucoidans obtained from *Ascophyllum nodosum* and *Fucusvesiculosus,* is observed at concentrations of 50–100 μg/mL [99]. Thus, the effective concentration of fucoidan extracts varies by the species of fucoidans. 

The molecular weight of fucoidan has been suggested as another important determinant of its anti-cancer effects in various types of malignancies [8,10,21]. However, there is no general agreement on the relationship between the potency of anti-cancer effects and the molecular weight fractions of fucoidan. It was believed that the pro-apoptotic activity of high molecular weight fucoidan (HMWF) was significantly higher than that of LMWF and middle molecular weight fucoidan (MMWF) [10,21,100]. However, other investigators suggest the opposite and state that LMWF has greater anti-angiogenic and anti-metastatic effects [101]. In fact, LMWF treatment suppressed tumor growth in a dose-dependent manner in xenograft mice implanted with human BC cells (T24 cells) [28]. Furthermore, it is important to note that the molecular weight of fucoidan in the serum remains unchanged; however, molecular weight of fucoidan isolated from the urine is significantly lower than that of the ingested form [102]. This information is important to discuss the difference in anti-cancer effects of fucoidan in BC. Moreover, although detailed information on absorption, distribution, metabolism, and extraction of fucoidan in human subjects is not fully understood, several reports have shown that fucoidan is detected in the serum/plasma of healthy human volunteers after oral administration of this compound [102,103]. Thus, oral intake of crude fucoidan is recognized as a useful mode of administration in human subjects. However, it is generally agreed upon that the absorption rate of orally administered fucoidan is dependent on its molecular weight. Correspondingly, LMWF shows a better absorption rate than MMWF or HMWF in an in vivo study [10,104]. 

On the other hand, we should note an important fact that the criteria of molecular weights are not uniform across studies. For example, although several investigators have defined <10 kDa as LMWF, 10–10,000 kDa as MMWF, and >10,000 kDa as HMWF [10,100], another study classifies 10–50 kDa as LMWF, 50–100 kDa as MMWF, and >100 kDa as HMWF [21]. 

### 7.2. Future Directions

As mentioned above, various new treatment strategies, such as fucoidan-based combination therapies and nanoparticles have been suggested and developed using in vivo and in vitro studies. In recent years, immune therapies have been used as standard treatment methods in various types of malignancies, including BC, and many investigators have paid special attention to the development and modification of this therapy [105,106,107]. Fucoidans play significant roles in mutation of dendritic cells, activity of T and B lymphocytes, macrophages, and natural killer cells, and differentiation of macrophages in different experimental models, including cancer xenograft models [12,99,108,109,110]. Importantly, such an immune system is closely associated with malignant potential and outcome of BC cells [111,112,113,114]. Additionally, immune therapy is currently recognized as a major treatment in patients with BC [115,116]. Based on these facts, fucoidan may enhance the anti-cancer effects of immune therapy as an immunomodulator in BC. However, no in vivo studies have clarified this hypothesis yet. On the other hand, while discussing the immunomodulatory effects of fucoidan, it is noteworthy to remember that its effects are dependent on the species from which it is extracted [99]. For example, the effect of activating the function of NK cells, T lymphocytes, and dendritic cells by fucoidan from *Macrocystis pyrifera* is stronger than those from others, such as *Ascophyllum nodosum*, *Undaria pinnatifida*, and *Fucus vesiculosus* [99]. Therefore, we emphasize that well-designed clinical studies are necessary to ascertain the anti-cancer effects of fucoidan as an immunomodulator in BC patients. 

Currently, photodynamic therapy and thermal therapy are emerging as new treatment tools for patients with malignancies [117,118]. Furthermore, photodynamic and photothermal therapy based on nanoparticles have been suggested as novel anti-cancer treatments [119,120]. Moreover, the anti-cancer effects and molecular mechanisms of photothermal therapy using photosensitive polypyrrole nanoparticles and fucoidan have been reported in 2020 [121]. Thus, the development of new treatment strategies using fucoidan-based anti-cancer agents is expected in the near future. On the other hand, regulation of ROS and VEGF are associated with the pharmacological activity of photothermal therapy using photosensitive polypyrrole nanoparticles and fucoidan [121]. It is well known that both ROS and VEGF play crucial roles in tumor growth, progression, and survival in cancer patients [52,122,123,124]. Therefore, we agree with the opinion that this regimen may become a potential and promising treatment for many types of malignancies, including BC. 

Currently, in most of the clinical trials on fucoidan, fucoidan is administered orally [125,126]. In contrast, a phase I clinical trial on imaging examination following intravenous injection of 99mTc-fucoidan has been reported [127]. Interestingly, this study showed that 99mTc-fucoidan did not have any drug-related adverse events [127]. Unfortunately, there is no clinical trial on the anti-cancer effects and safety of intravenously injected fucoidan in cancer patients. Conversely, although an effective fucoidan dose is dependent on the type of cancer, the effective dose for BC seems to be lower than that for other cancers [15,128]. For example, the effective dose of fucoidan derived from *Fucus vesiculosus* in hepatocellular carcinoma cell line and that of fucoidan supplied by Sigma-Aldrich Chemical Co. in prostate cancer cell line was reported to be 1,000 μg/mL. Thus, anti-cancer effects of fucoidan may be stronger in BC compared to those in the other types of cancers. In addition, there is the possibility that intravesical administration of fucoidan can suppress the recurrence of non-muscle invasive BC. We think a treatment strategy based on non-oral administration of fucoidan may have stronger biological effects in patients with malignancies including BC. 

## 8. Conclusions

We reviewed the anti-cancer effects of fucoidan and fucoidan-based treatment strategies and their detailed molecular mechanisms. Accordingly, fucoidan is speculated to have clinical application and is a potential therapeutic for patients with malignancies, including BC. On the other hand, we should note that its biological and pharmacological activities are dependent on many internal and external factors, such as the species it is extracted from and its molecular weight. Unfortunately, there are very few clinical trials, including comparative and randomized studies in patients with BC. However, it is difficult to plan clinical trials with large populations in BC patients because basic information regarding the pharmacological characteristics of fucoidan, such as absorption, distribution, metabolism, and pharmacokinetics based on the molecular weight, is still unavailable. Nevertheless, we emphasize that fucoidan is a much sought-after compound, owing to its low in vivo toxicity, including that in humans. Lastly, we suggest that well-designed preclinical and clinical trials are needed to investigate the anti-cancer effects and safety of fucoidan-based therapies in patients with BC. 

## Figures and Tables

**Table 1:** Anticancer effects of fucoidans in bladder cancer cells.

Pathological Feature	Cell Line	Design	Species	Type	Dose	Year/References
Tumor growth	5637	In vitro	Not tested	Crude	50–400 *	2014/[26]
	T24	In vitro	Not tested	Crude	100–150 *	2014/[27]
	T24	In vivo	*Sargassum hemiphyllum*	LMWF	160–300 **	2015/[28]
	EJ	In vitro	*Fucusvesiculosus*	Crude	50–150 *	2015/[29]
	5637	In vitro	*Fucusvesiculosus*	Crude	25–100 *	2017/[30]
Apoptosis	T24	In vitro	Not tested	Crude	50–150 *	2014/[27]
	EJ	In vitro	*Fucusvesiculosus*	Crude	50–150 *	2015/[29]
	5637	In vitro	*Fucusvesiculosus*	Crude	50–100 *	2017/[30]
Migration/invasion	5637	In vitro	Not tested	Crude	100 *	2014/[26]
	T24	In vitro	Not tested	Crude	100 *	2014/[26]
	T24	In vitro	*Sargassum hemiphyllum*	LMWF	25–100 *	2015/[28]
Angiogenesis	T24	In vitro	*Sargassum hemiphyllum*	LMWF	25–100 *	2015/[28]

LMWF: low molecular weight fucoidan (760 Da), Doses: * μg/mL, ** mg/kg/day.

**Table 2:** Fucoidan-induced changes of cell survival-related molecules.

Molecules	Change	Cell Line	Species	Year/Reference
Akt/PI3K	↓	5637	Not tested	2014/[26]
	↓	5637	*Fucusvesiculosus*	2017/[30]
Bax	↑	T24	Not tested	2014/[27]
	↑	5637	*Fucusvesiculosus*	2017/[30]
Bcl-2	↓	T24		2014/[27]
	↓	5637	*Fucusvesiculosus*	2017/[30]
Bid	↓	T24	Not tested	2014/[27]
truncated Bid	↑	T24	Not tested	2014/[27]
Caspase-3	↑	T24	Not tested	2014/[27]
Caspase-8	↑	T24	Not tested	2014/[27]
Caspase-9	↑	T24	Not tested	2014/[27]
cIAP-1	↓	T24	Not tested	2014/[27]
cIAP-2	↓	T24	Not tested	2014/[27]
DR4	NC	T24	Not tested	2014/[27]
DR5	↑	T24	Not tested	2014/[27]
Fas	↑	T24	Not tested	2014/[27]
XIAP	↓	T24	Not tested	2014/[26]

PI3K: phosphoinositide 3-kinase, Bax: Bcl-2 associated protein, Bcl-2: B-cell lymphoma 2, Bid: BH3 interacting domain death agonist, cIAP: cellular inhibitor of apoptosis, DR: death receptor, XIAP: X-chromosome-linked inhibitor of apoptosis.

**Table 3:** Fucoidan-induced changed of cell-cycle-related molecules.

Molecules	Change	Cell Line	Year/Reference
Cdk2	↓	5637	2014/[26]
	↓	T24	2014/[27]
	↓	RJ	2015/[29]
Cdk4	↓	5637	2014/[26]
	↓	T24	2014/[27]
	↓	RJ	2015/[29]
Cdk6	↓	T24	2014/[27]
	↓	RJ	2015/[29]
cyclin D1	↓	56372	2014/[26]
	↓	T24	2014/[27]
	↓	RJ	2015/[29]
cyclin E	↓	5637	2014/[26]
	↓	T24	2014/[27]
	↓	RJ	2015/[29]
E2F-1	No change	T24	2014/[27]
	No change	RJ	2015/[29]
E2F-4	No change	T24	2014/[27]
	No change	RJ	2015/[29]
p21	↑	T24	2014/[28]
	No change	RJ	2015/[29]
p21WAF1	↑	5637	2014/[26]
p27	No change	T24	2014/[27]
	No change	RJ	2015/[29]
pRb	↓	T24	2014/[27]
	↓	RJ	2015/[29]

Cdk; cyclin-dependent kinase, Rb, retinoblastoma.

**Table 4:** Increased anti-cancer effects of fucoidan combined with chemotherapeutic agents.

Agents	Type of Malignancy	*Species*	Design	Reference
CDDP	Breast cancer	*Cladosiphonnavae-caledoniae*	In vitro	[64]
	Breast cancer	*Fucusvesicluosus*	In vitro	[62]
	Head and neck cancer	*Fucusvesicluosus*	In vitro	[61]
	Lung cancer	*Fucusvesiculosus*	Both	[63]
	Colorectal cancer	*Sargassum hemiphyllum* *	Both	[12]
GEM	Breast cancer	*Fucusvesiculosus*	In vitro	[65]
	Tongue	*Fucusvesiculosus*	In vitro	[65]
	Melanoma	*Fucusvesiculosus*	In vitro	[65]
	Ovarian cancer	*Fucusvesiculosus*	In vitro	[65]
	Breast cancer	*Undaria pinnatifida*	In vitro	[65]
	Tongue	*Undaria pinnatifida*	In vitro	[65]
	Uterine sarcoma	*Undaria pinnatifida*	In vitro	[61]
	Uterine carcinosarcoma	*Undaria pinnatifida*	In vitro	[61]
PTX	Breast cancer	*Cladosiphonnavae-caledoniae*	In vitro	[64]
	Cervical cancer	*Undaria pinnatifida*/*Fucusvesiculosus*	In vitro	[65]
	Endometrial cancer	*Undaria pinnatifida*/*Fucusvesiculosus*	In vitro	[65]
	Melanoma	*Undaria pinnatifida*/*Fucusvesiculosus*	In vitro	[65]
	Ovarian cancer	*Undaria pinnatifida*/*Fucusvesiculosus*	In vitro	[65]
	Prostate cancer	*Undaria pinnatifida*/*Fucusvesiculosus*	In vitro	[65]
	Tongue cancer	*Undaria pinnatifida*/*Fucusvesiculosus*	In vitro	[65]

CDDP: cisplatin, GEM: gemcitabine, PTX: paclitaxel.
